# Effects of an exogenous ketone ester using multi-omics in skeletal muscle of aging C57BL/6J male mice

**DOI:** 10.3389/fnut.2022.1041026

**Published:** 2022-11-15

**Authors:** Brandon M. Roberts, Sarah E. Deemer, Daniel L. Smith, James A. Mobley, Nicolas Musi, Eric P. Plaisance

**Affiliations:** ^1^Department of Human Studies, Division of Molecular and Translational Biomedicine, University of Alabama at Birmingham, Birmingham, AL, United States; ^2^Department of Kinesiology, Health Promotion, and Recreation, University of North Texas, Denton, TX, United States; ^3^Department of Nutrition Sciences, Division of Molecular and Translational Biomedicine, University of Alabama at Birmingham, Birmingham, AL, United States; ^4^Department of Anesthesiology and Perioperative Medicine, Division of Molecular and Translational Biomedicine, University of Alabama at Birmingham, Birmingham, AL, United States; ^5^Barshop Institute for Longevity and Aging Studies, University of Texas Health Science Center San Antonio, San Antonio, TX, United States; ^6^San Antonio Geriatric Research, Education, and Clinical Center, San Antonio, TX, United States

**Keywords:** skeletal muscle, ketone ester, nutrition, sarcopenia, proteomics, lipidomics

## Abstract

Exogenous ketone ester supplementation provides a means to increase circulating ketone concentrations without the dietary challenges imposed by ketogenic diets. Our group has shown that oral R,S-1,3, butanediol diacetoacetate (BD-AcAc_2_) consumption results in body weight loss or maintenance with moderate increases in circulating ketones. We have previously shown a diet consisting of 25% BD-AcAc_2_ can maintain lean body mass (LBM) and induce fat mass (FM) loss in young, healthy male mice, but the underlying mechanisms are still unknown. Therefore, the purpose of this study was to determine if a diet consisting of 25% BD-AcAc_2_ (ketone ester, KE) would alter body composition, transcriptional regulation, the proteome, and the lipidome of skeletal muscle in aged mice. We hypothesized that the KE group would remain weight stable with improvements in body composition compared to controls, resulting in a healthy aging phenotype. Male C57BL/6J mice (*n* = 16) were purchased from Jackson Laboratories at 72 weeks of age. After 1 week of acclimation, mice were weighed and randomly assigned to one of two groups (*n* = 8 per group): control (CON) or KE. A significant group by time interaction was observed for body weight (*P* < 0.001), with KE fed mice weighing significantly less than CON. FM increased over time in the control group but was unchanged in the KE group. Furthermore, LBM was not different between CON and KE mice despite KE mice weighing less than CON mice. Transcriptional analysis of skeletal muscle identified 6 genes that were significantly higher and 21 genes that were significantly lower in the KE group compared to CON. Lipidomic analysis of skeletal muscle identified no differences between groups for any lipid species, except for fatty acyl chains in triacylglycerol which was 46% lower in the KE group. Proteomics analysis identified 44 proteins that were different between groups, of which 11 were lower and 33 were higher in the KE group compared to CON. In conclusion, 72-week-old male mice consuming the exogenous KE, BD-AcAc_2_, had lower age-related gains in body weight and FM compared to CON mice. Furthermore, transcriptional and proteomics data suggest a signature in skeletal muscle of KE-treated mice consistent with markers of improved skeletal muscle regeneration, improved electron transport chain utilization, and increased insulin sensitivity.

## Introduction

Ketogenic diets (KDs) improve or maintain metabolic function and attenuate age-related increases in adiposity and bodyweight, presumably by increasing circulating ketone concentrations in both animal models and humans (1, 2). Clinical studies have demonstrated the beneficial effect of KDs as an efficient nutritional strategy in the treatment of obesity (3–5). This effect occurs not only in modifying body weight and body composition but also in modulating epigenetic markers, as well as circulating levels of myokines and circulating levels of cytokines and markers of oxidative stress and this effect appears to be induced by the action of ketone bodies synergistically with the weight loss induced by the VLCKD treatment (6–8). In mice, KDs have been shown to extend longevity and healthspan by decreasing oxidative and endoplasmic reticulum (ER) stress resulting in lower protein turnover in skeletal muscle, which may allow greater maintenance of muscle mass and function with both age and disease (9, 10). These findings have practical implications in humans, but by nature of the requirement for extensive carbohydrate restriction and high fat content (often > 75%), KDs present adherence challenges and may not be a long-term strategy for all humans.

Exogenous ketone ester (KE) supplementation provides a means to increase circulating ketone concentrations without the dietary challenges imposed by KDs. Ketone esters have been used in many forms such as R,S-1,3-butanediol diacetoacetate (BD-AcAc_2_, ketone diester), *D*-β-hydroxybutyrate-(R)-1,3 butanediol (ketone monoester), and more recently Bis-hexanoyl (R)-1,3-butanediol (BH-BD) (11–13). Studies in animal models show that oral BD-AcAc_2_ consumption results in body weight loss or maintenance with moderate increases in circulating ketones (0.5–1.0 mM) (14–16). Recently, we examined concentration-dependent effects of BD-AcAc_2_ on body weight, adiposity, energy intake, and energy expenditure in lean mice showing that on an *ad libitum* basis, mice consuming a 25% (by kcals) KE diet consumed the same amount of food as an *ad libitum* fed control group, but had a significant difference in body weight (BW) and fat mass (FM), and maintained lean body mass (LBM) (17). Furthermore, after adjustment for LBM and FM, there was no difference in resting energy expenditure (REE) compared to control (17). Our group has also shown that BD-AcAc_2_ induces loss of BW and FM in diet-induced obesity without changes in LBM or changes in skeletal muscle thermogenic activity (18). Furthermore, when housed in thermoneutral conditions, mice consuming BD-AcAc_2_ decreased body weight resulting in lower adiposity. The decrease in body weight observed in KE-fed mice transpired without an increase in REE or TEE (19). These findings suggest that a diet consisting of 25% BD-AcAc_2_ can maintain LBM and induce FM loss in young, healthy male mice, but the underlying mechanisms are still unknown.

Despite extensive evidence of reductions or maintenance of body weight and adiposity, little is known regarding the effects of BD-AcAc_2_ on skeletal muscle. Previously, we found that a KE diet does not produce transcriptional changes consistent with mitochondrial biogenesis or respiration (18). Therefore, the purpose of this study was to determine if a diet consisting of 25% BD-AcAc_2_ would alter body composition, transcriptional regulation, the proteome, and the lipidome of skeletal muscle in aged B6 mice. We hypothesized that the KE group would remain weight stable with improvements in body composition (higher LBM and lower FM) compared to controls resulting in a healthy aging phenotype.

## Materials and methods

### Study design and diets

Male C57BL/6J mice (*n* = 16) were purchased from Jackson Laboratories (Bar Harbor, ME, USA) at 72 weeks of age and acclimated for 7 days. At 73 weeks of age, mice were weighed and randomly assigned control (CON, *n* = 8) or KE (KE, *n* = 8) diet. Mice were fed *ad libitum* for 9 weeks. The KE diet (Dyets Inc., Bethlehem, PA, #104425) contained 25% kcals from BD-AcAc_2_, which replaced 25% of carbohydrate energy in the CON diet (Dyets Inc., #104419) with fat and protein content equal between the diets. Each diet was 3.7 kcal/g and details of the individual diets (CON and KE) are described in Deemer et al. (17). The UAB Institutional Animal Care and Use Committee (IACUC) approved the investigation.

### Husbandry

Mice were single-housed and maintained on a standard 12:12 light-dark cycle. All animals had access to *ad libitum* water and food throughout the study. Food intake and body weight were recorded every day, and fresh food was given each day at the same time. Two body composition (fat and lean body mass) measurements were completed in the University of Alabama at Birmingham (UAB) Small Animal Phenotyping Core on day 33 and day 66 of the study by quantitative magnetic resonance (QMR; EchoMedical MRI, Houston, TX, USA). Due to COVID-19 shutdowns body composition was not obtained at baseline.

### RNA isolation and nanostring analysis

Tissue samples were flash frozen in liquid nitrogen at the time of dissection and stored at −80°C. RNA from vastus lateralis was isolated according to manufacturer instructions (RNEasy Mini Kit; Qiagen; Germany). All procedures occurred at room temperature. Briefly, an approximately 30 mg sample was homogenized in Buffer RLT with metal beads using a bead mill (FisherbrandTM Bead Mill Homogenizer) for 2 min at 5 m/s. Lysate was centrifuged for 3 min and the supernatant was transferred to a new microcentrifuge tube. A 1:1 by volume of 70% ethanol was added to the supernatant and mixed by trituration. Approximately 700 μL of sample was transferred to the Qiagen spin column and isolation of mRNA was completed per manufacturer instructions. The A260/A280 ratio was measured to quantify mRNA purity (Thermo ScientificTM NanoDropTM Lite Spectrophotometer) and isolated mRNA was concentrated to 15 ng/μL prior to NanoString analysis. Concentrated mRNA was analyzed using a 770-plex Mouse PanCancer Pathways kit (nCounter; NanoString, Seattle, WA, United States) and raw image counts (RCC files) were obtained using a SPRINT Profiler (NanoString) following a 16-h hybridization assay at 65°C.

Data was analyzed by ROSALIND^®^,^1^ with a HyperScale architecture developed by ROSALIND, Inc., (San Diego, CA, USA). Read Distribution percentages, violin plots, identity heatmaps, and sample MDS plots were generated as part of the QC step. Normalization, fold changes, and *p*-values were calculated using criteria provided by Nanostring. Housekeeping probes to be used for normalization are selected based on the geNorm algorithm as implemented in the NormqPCR R library (20). Fold changes and *p*-values are calculated using the fast method as described in the nCounter^®^ Advanced Analysis 2.0 User Manual. *P*-value adjustment is performed using the Benjamini-Hochberg method of estimating false discovery rates (FDR).

#### Proteomics

Proteomics analysis was carried out as previously referenced with minor differences (21). Proteomics samples were compared in the control (*n* = 7) and KE groups (*n* = 6). All protein extracts were obtained using T-PER™ Mammalian Protein Extraction Reagent (Thermo Fisher Scientific, Cat# 78510) containing Halt Protease Inhibitor Cocktail (Thermo Fisher Scientific, Cat# 78429) and Dounce-homogenized in lysis buffer, 20–30 strokes per sample, and centrifuged (∼12 Kg) for 10 min at 4*^o^*C to remove debris. Protein extracts were then quantified using a Pierce BCA Protein Assay Kit (Thermo Fisher Scientific, Cat# PI23225). Forty (40) μg of protein was diluted in 35 μL of NuPAGE LDS sample buffer (1x final conc., Invitrogen, Cat# NP0007) for each sample. Proteins were then reduced with dithiothreitol (DTT) and denatured at 70°C for 10 min prior to loading onto Novex NuPAGE 10% Bis-Tris protein gels (Invitrogen, Cat.# NP0315BOX) and separated (35 min at 200 V). Gels were stained overnight with Novex Colloidal Blue Staining kit (Invitrogen, Cat# LC6025). Following de-staining, each lane was cut into 6-molecular weight (MW) fractions and equilibrated in 100 mM ammonium bicarbonate (AmBc). Each gel plug was then digested overnight with Trypsin Gold, Mass Spectrometry Grade (Promega, Cat# V5280) following the manufacturer’s instructions. Peptide extracts were reconstituted in 0.1% formic acid/ddH_2_O at 0.1 μg/μL. Peptide digests (8 μL each) were injected onto a 1,260 Infinity nHPLC stack (Agilent Technologies), and separated using a 75 micron I.D. x 15 cm pulled tip C-18 column (Jupiter C-18 300 Å, 5 micron, Phenomenex).

The XCalibur RAW files were collected in profile mode, centroided and converted to MzXML using ReAdW v. 3.5.1. Mgf files were then created using MzXML2Search (included in TPP v. 3.5) for all scans. The data was searched using SEQUEST (Thermo Fisher Scientific), which was set for three maximum missed cleavages, a precursor mass window of 20 ppm, trypsin digestion, variable modification C at 57.0293, and M @ 15.9949 as a base setting. Searches were performed with redundant sequences removed on the *mus musculus* specific subset of the UniProt100 database. The list of peptide IDs generated based on SEQUEST search results were filtered using Scaffold (Protein Sciences, Portland, OR, USA). The cut-off values included a minimum peptide length of > 5 AA’s, with no MH + 1 charge states, with peptide probabilities of > 80% C.I., and with the number of peptides per protein ≥ 2. The protein probabilities were set to a > 99.0% C.I. and an FDR < 1.0. Scaffold incorporates the two most common methods for statistical validation of large proteome datasets, the false discovery rate (FDR) and protein probability (22–24). Relative quantification across experiments was then performed *via* spectral counting (25, 26). Spectral count abundances were then normalized between samples (27).

For protein abundance ratios determined, we set a 1.5-fold change as the threshold for significance, determined empirically by analyzing the inner-quartile data from the control experiments using ln-ln plots, where the Pierson’s correlation coefficient (R) is 0.98, and > 95–99% of the normalized intensities fell between the set fold changes. In each case, both tests (*t*-test and fold change) were required to pass in order to be considered significant. All multivariate analyses, including 2D HCA HeatMaps and PCA plots were carried out using Qlucore Omics Explorer (Qlucore, Lund Sweden). Gene ontology assignments and pathway analysis were carried out using MetaCore (GeneGO Inc., St. Joseph, MI, USA). Interactions identified within MetaCore are manually correlated using full text articles. Detailed algorithms have been described previously (28, 29).

#### Lipidomics

Lipid species were analyzed using multidimensional mass spectrometry-based shotgun lipidomic analysis (30). In brief, muscle homogenates containing 0.6 mg of protein (quantified by Pierce BCA assay) was transferred to a disposable glass test tube. A premixture of lipid internal standards (IS) was added prior to conducting lipid extraction for quantification of the targeted lipid species. Lipid extraction was performed using a modified Bligh and Dyer procedure (31), and each lipid extract was reconstituted in chloroform:methanol (1:1, *v:v*) at a volume of 400 μL/mg protein.

For shotgun lipidomics, lipid extract was further diluted to a final concentration of ∼500 fmol total lipids per μL. Mass spectrometric analysis was performed on a triple quadrupole mass spectrometer (TSQ Altis, Thermo Fisher Scientific, San Jose, CA, USA) and a Q Exactive mass spectrometer (Thermo Scientific, San Jose, CA, USA), both of which were equipped with an automated nanospray device (TriVersa NanoMate, Advion Bioscience Ltd., Ithaca, NY, USA) as described (32). Identification and quantification of lipid species were performed using an automated software program (33, 34). Data processing (e.g., ion peak selection, baseline correction, data transfer, peak intensity comparison and quantitation) was performed as described (34). The result was normalized to protein content (nmol lipid/mg protein or pmol lipid/mg protein).

#### Statistical analysis of body weight, food intake, and body composition

Graphical and formal statistical tests performed using PROC UNIVARIATE revealed that all variables were normally distributed. Differences within and between groups were analyzed using a GROUP x TIME repeated measures analysis of variance (ANOVA) in SAS (version 9.4m7, Cary, NC, USA). For body weight and food intake, weekly mean values of mice within a group were compared over the 9 weeks. Body composition variables were compared between day 33 and day 66 between and within groups. Due to COVID-19 restrictions, a baseline body composition measurement was not performed. Tissue weights between groups were analyzed by one-way ANOVA. Tukey-Kramer *post hoc* testing was used to explore significant differences determined by RMANOVA. Significance was set *a priori* at *P* < 0.05. Data are expressed as means ± standard error of the mean (SEM).

## Results

### Body weight, muscle weight, body composition, and food intake

**FIGURE 1:**
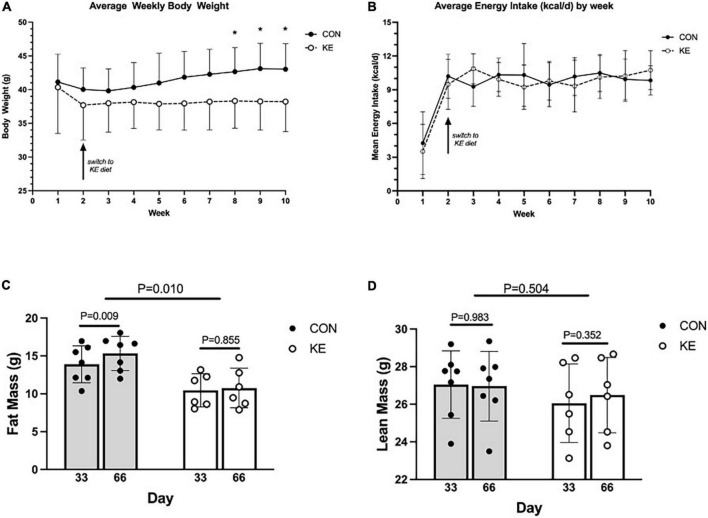
**(A)** Weekly mean body weight of ketone ester (KE) mice was significantly less than control (CON) mice only during the last 3 weeks of the dietary intervention. **(B)** Weekly energy intake was similar between CON and KE groups. **(C)** Fat mass measured by quantitative magnetic resonance (QMR) was greater in CON compared to KE mice, and increased from day 33 to day 66 in CON mice only. **(D)** Lean mass measured by QMR was not different between CON and KE mice despite KE mice weighing less than CON mice.

Groups were not significantly different in body weight at baseline. There was a significant group by time interaction in the overall analysis for body weight (*P* < 0.001), with KE fed mice weighing significantly less than CON at endpoint. However, these differences were not significant after *post hoc* adjustments for multiple testing. Interestingly, there was a within-group increase in body weight in the CON group in week 2 compared to week 8 (*P* < 0.01), week 9 (*P* < 0.001), and week 10 [(*P* < 0.001) Figure 1A]. There were no between or within group changes in energy intake (kcal/day) (Figure 1B). For body composition, there was an overall group by time interaction (*P* < 0.01), which was driven by a within-group increase in FM in the CON group (*P* < 0.01) while there was no significant within-group change in the KE group (Figures 1C,D). There were also no significant differences in LBM, muscle wet weight of vastus lateralis, gastrocnemius, soleus, and tibialis anterior between groups (data not shown).

### Gene expression

A broad approach was employed to compare gene expression in vastus lateralis between groups using the ROSALIND gene array. This analysis was completed at the endpoint (week 83) of the study. Of the 773 genes analyzed, 6 were significantly higher in the KE group compared CON (Table 1). 23 genes were also significantly lower in the KE group compared to CON (Table 2).

**TABLE 1:** Genes that were significantly higher in ketone ester (KE) compared to control (CON).

Gene	Gene name	Cellular process	Fold change	*p*-value
*Gadd45a*	Growth arrest and DNA damage inducible alpha 45	regulation of cell cycle	1.86	0.006
*Pla2g4e*	Phospholipase A2 group IVE	cytosol phospholipase	1.23	0.038
*Runx1*	RUNX family transcription factor 1	transcription regulator	1.69	0.040
*Tnc*	Tenascin C	extracellular matrix protein	1.44	0.032
*Cdc14a*	Cell division cycle 14A	protein phosphatase	1.26	0.027
*Bax*	BCL2 associated X, apoptosis regulator	mitochondrial membrane protein	1.13	0.021

**TABLE 2:** Genes that were significantly lower in ketone ester (KE) compared to control (CON).

Gene	Gene name	Cellular process	Fold change	*p*-value
*Pim1*	Pim-1 proto-oncogene, serine/threonine kinase	transmembrane glycoprotein	−1.56279	0.049
*Fgfr4*	Fibroblast growth factor receptor 4	growth factor	−1.37815	0.049
*Prkcg*	Protein kinase C gamma	transferase activity	−1.29662	0.032
*Smad3*	SMAD family member 3	transcription regulator	−1.24761	0.023
*Irak2*	Interleukin 1 receptor associated kinase 2	signal transduction	−1.22257	0.041
*Cbl*	Cbl proto-oncogene	ubiquitin protein ligase	−1.18758	0.048
*Igfbp3*	Insulin like growth factor binding protein 3	protease inhibitor	−1.15034	0.049
*Tsc2*	TSC complex subunit 2	GTPase activator	−1.13114	0.015
*Axin1*	Axin 1	angiogenesis	−1.10821	0.029
*Mycn*	MYCN proto-oncogene, BHLH transcription factor	transcription regulator	−1.82609	0.021
*Itga3*	Integrin subunit alpha 3	membrane protein	−1.46154	0.002
*Rac3*	Rac family small GTPase 3	transcription regulator	−1.37600	0.004
*Mecom*	MDS1 And EVI1 complex locus protein EV1	transcription regulator	−1.26208	0.007
*Rps6ka5*	Ribosomal protein S6 kinase A5	protein signaling	−1.22216	0.015
*Tgfb3*	Transforming growth factor beta 3	growth factor	−1.17609	0.006
*Tsc1*	TSC complex subunit 1	protein signaling	−1.13642	0.001
*Camk2b*	Calcium/calmodulin dependent protein kinase II beta	calcium signaling	−1.12545	0.001
*Traf7*	TNF receptor associated factor 7	ubiquitin protein ligase	−1.10585	0.003
*Prom1*	Prominin 1	cholesterol binding	−1.37095	0.009
*Hdac6*	Histone deacetylase 6	transcription regulator	−1.22481	0.036
*Dvl2*	Disheveled segment polarity protein 2	cytoplasmic protein	−1.21628	0.012
*Phf6*	PHD finger protein 6	zinc finger protein	−1.16103	0.038
*Ptpn11*	Protein tyrosine phosphatase non-receptor type 11	protein phosphatase	−1.11103	0.016

### Lipidomics

Lipidomics was performed on vastus lateralis in CON (*n* = 7) and KE groups (*n* = 6). There were no differences between groups for any lipid species, except for fatty acyl chains in triacylglycerol (nmol/mg protein) which was 46% lower in the KE group compared to CON (*P* = 0.05, Table 3).

**TABLE 3:** Skeletal muscle lipidomic analysis.

Lipid	CON	KE	*p*-value
Spingomyelin (pmol/mg protein)	1267.8 ± 243.9	1255.4 ± 139.0	0.915
Phosphatidlycholine (nmol/mg protein)	49.5 ± 9.2	47.0 ± 3.7	0.534
Lyso-Phosphatidlycholine (nmol/mg protein)	1611.5 ± 304.4	1920.8 ± 393.0	0.138
Phosphatidylethanolamine (nmol/mg protein)	43.2 ± 5.2	48.0 ± 8.9	0.253
Lysophosphatidylethanolamine (pmol/mg protein)	803.7 ± 160.1	845.1 ± 159.2	0.650
Ceramide (pmol/mg protein)	272.5 ± 41.3	272.2 ± 52.2	0.991
Cardiolipin (nmol/mg protein)	4.0 ± 0.9	3.7 ± 0.5	0.501
Lysocardiolipin (nmol/mg protein)	17.1 ± 7.3	25.8 ± 8.4	0.070
Phosphatidylinositol (nmol/mg protein)	5.0 ± 0.8	4.8 ± 0.7	0.523
Phosphatidylserine (nmol/mg protein)	2.5 ± 0.5	2.4 ± 0.2	0.620
Phosphatidlyglycerol/Bis (monoacylglycerol) phosphate	2.0 ± 0.2	1.9 ± 0.3	0.317
Phosphatidic acid (pmol/mg protein)	31.4 ± 6.4	30.8 ± 2.1	0.846
Triacylglycerol (nmol/mg protein)	813.1 ± 303.4	592.4 ± 256.3	0.188
Fatty acyl chains in triacylglycerol (nmol/mg protein)	2452.2 ± 914.2	1787.3 ± 771.2	**0.050**
*De novo fatty acids	1742.7 ± 637.3	1245.9 ± 512.9	0.155

*De novo fatty acids were examined cumulatively as 14:0, 14:1, 16:0, 16:1, 18:0, 18:1, 22:0, 22:1. Values are means ± SD. Significant was set *a priori* at *P* < 0.05. Data are represented as Mean ± SD. The bold value represents the statistical significance.

### Proteomics

There were 1,020 protein IDs with > 99% C.I. and < 1% FDR from the initial analysis. Proteomics was performed on the gastrocnemius in CON (*n* = 7) and KE groups (*n* = 6). Many proteins identified were in low enough abundance and presented with a fair amount of heterogeneity “zero” values across each group and were therefore eliminated from the analysis. Of those proteins that were more homogeneous, 545 high confidence hits were observed in at least 3 or more specimens per group with a signal over zero. A non-parametric statistical analysis was then applied to these remaining proteins along with a fold change of > 1.5 or < −1.5, which led to 44 proteins that were statistically changed in abundance in the KE compared to CON. Of those, 11 were significantly higher in KE compared to CON (Table 4) and 33 were significantly lower (Table 5) in KE compared to CON.

**TABLE 4:** Proteomic differences in mouse muscle tissues: Ketone ester (KE) vs. control (CON) 11 proteins were significantly higher in KE compared to CON.

					Statistics			
UniProtKB names	Gene name	Prot Acc#	GeneID	Avg (C)	Avg (KE)	P	SAM	Fold (KE/C)
COP9 signalosome complex subunit 4	COP9 signalosome subunit 4 (Cops4)	O88544	26891	1.0	2.7	0.002	3.26	2.63
Serine/threonine-protein kinase mTOR	mechanistic target of rapamycin (Mtor)	Q9JLN9	56717	1.5	2.9	0.016	1.33	1.96
Quinone oxidoreductase	crystallin, zeta (Cryz)	P47199	12972	2.7	4.9	0.007	0.93	1.84
Electron transfer flavoprotein-ubiquinone oxidoreductase	electron transferring flavoprotein, dehydrogenase (Etfdh)	Q921G7	66841	3.8	6.8	0.004	0.90	1.80
Thioredoxin-like protein 1	thioredoxin-like 1 (Txnl1)	Q8CDN6	53382	1.3	2.3	0.012	0.97	1.76
Probable ubiquitin carboxyl-terminal hydrolase FAF-X	ubiquitin specific peptidase 9, X chromosome (Usp9x)	P70398	22284	8.8	15.2	0.010	0.75	1.72
Protein Col6a3	collagen, type VI, alpha 3 (Col6a3)	J3QQ16	12835	4.8	8.2	0.026	0.73	1.69
Voltage-dependent anion-selective channel protein 1	voltage-dependent anion channel 1 (Vdac1)	Q60932	22333	2.8	4.7	0.048	0.56	1.67
Mutant fibrillin-1	fibrillin 1 (Fbn1)	O88840	14118	4.0	6.7	0.049	0.62	1.66
Protein Tnxb	tenascin XB (Tnxb)	O35452	81877	14.4	23.8	0.012	0.79	1.66
ENH isoform 3a	PDZ and LIM domain 5 (Pdlim5)	D9J303	56376	8.5	12.8	0.021	0.64	1.50

**TABLE 5:** Proteomic differences in mouse muscle tissues: Ketone ester (KE) vs. control diets 33 proteins were significantly lower in KE compared to CON.

						Statistics		
UniProtKB names	Gene name	Prot Acc#	GeneID	Avg (C)	Avg (KE)	P	SAM	Fold (KE/C)
Serine protease inhibitor A3K	serine (or cysteine) peptidase inhibitor, clade A, member 3K (Serpina3k)	P07759	20714	8.1	2.5	0.022	0.81	−3.27
Serine/threonine-protein phosphatase 2B	protein phosphatase 3, catalytic subunit, alpha isoform (Ppp3ca)	P63328	19055	3.8	1.3	0.017	0.88	−2.82
10 kDa heat shock protein	heat shock protein 1 (chaperonin 10) (Hspe1)	Q64433	15528	4.6	1.7	0.020	1.00	−2.66
Annexin A1	annexin A1 (Anxa1)	P10107	16952	5.1	2.0	0.029	0.84	−2.58
Alpha-2-macroglobulin	pregnancy zone protein (Pzp)	Q61838	11287	23.9	9.9	0.003	0.98	−2.40
Acyl-coenzyme A thioesterase 13	acyl-CoA thioesterase 13 (Acot13)	Q9CQR4	66834	8.6	3.8	0.041	0.53	−2.24
ES1 protein homolog, mitochondrial	DNA segment, Chr 10, Johns Hopkins University 81 expressed (D10Jhu81e)	Q9D172	28295	13.5	6.2	0.001	1.14	−2.15
Murinoglobulin-1	murinoglobulin 1 (Mug1)	P28665	17836	20.6	9.7	0.002	1.13	−2.13
Transgelin	transgelin (Tagln)	P37804	21345	5.1	2.4	0.043	0.58	−2.12
Gelsolin, isoform CRA_c	gelsolin (Gsn)	Q6PAC1	227753	3.9	1.9	0.016	0.74	−2.08
Dual specificity protein phosphatase 3	dual specificity phosphatase 3 (vaccinia virus phosphatase VH1-related) (Dusp3)	Q9D7 × 3	72349	8.1	3.9	0.004	1.01	−2.07
S-formylglutathione hydrolase	esterase D/formylglutathione hydrolase (Esd)	Q9R0P3	13885	2.7	1.3	0.010	1.05	−2.02
Vesicle-associated membrane protein	vesicle-associated membrane protein, associated protein A (Vapa)	Q9WV55	30960	6.5	3.3	0.030	0.67	−1.98
Galectin-1	lectin, galactose binding, soluble 1 (Lgals1)	P16045	16852	10.6	5.4	0.002	1.15	−1.98
Endoplasmin	heat shock protein 90, beta (Grp94), member 1 (Hsp90b1)	P08113	22027	5.7	3.0	0.007	0.89	−1.90
Glutathione S-transferase kappa 1	glutathione S-transferase kappa 1 (Gstk1)	Q9DCM2	76263	3.4	1.8	0.021	0.88	−1.84
6-phosphogluconolactonase	6-phosphogluconolactonase (Pgls)	Q9CQ60	66171	4.2	2.3	0.015	0.84	−1.83
Thioredoxin reductase 1	thioredoxin reductase 1 (Txnrd1)	Q9JMH6	50493	3.8	2.1	0.041	0.64	−1.82
EH domain-containing protein 2	EH-domain containing 2 (Ehd2)	Q8BH64	259300	5.8	3.2	0.038	0.62	−1.81
Smoothelin-like protein 1	smoothelin-like 1 (Smtnl1)	Q99LM3	68678	2.2	1.2	0.048	0.64	−1.79
Carboxylesterase 1C	carboxylesterase 1C (Ces1c)	P23953	13884	9.5	5.5	0.030	0.72	−1.72
Hemopexin	hemopexin (Hpx)	Q91 × 72	15458	6.5	3.8	0.042	0.53	−1.70
Alpha-1-antitrypsin 1-2	serine (or cysteine) preptidase inhibitor, clade A, member 1B (Serpina1b)	P22599	20701	12.6	7.5	0.019	0.82	−1.69
Annexin A2	annexin A2 (Anxa2)	P07356	12306	14.4	8.8	0.023	0.62	−1.64
Phosphorylase b kinase	phosphorylase kinase alpha 1 (Phka1)	P18826	18679	10.3	6.4	0.030	0.60	−1.62
Fatty acid-binding protein, adipocyte	fatty acid binding protein 4, adipocyte (Fabp4)	P04117	11770	36.9	23.0	0.029	0.59	−1.60
Alpha-1-antitrypsin 1-3	serine (or cysteine) peptidase inhibitor, clade A, member 1C (Serpina1c)	Q00896	20702	11.1	7.0	0.005	0.86	−1.59
Ubiquitin-conjugating enzyme E2 variant 2	ubiquitin-conjugating enzyme E2 variant 2 (Ube2v2)	Q9D2M8	70620	4.2	2.7	0.044	0.72	−1.55
Citrate lyase subunit beta-like protein	citrate lyase beta like (Clybl)	Q8R4N0	69634	2.8	1.8	0.020	0.68	−1.55
Ras-related protein Rab-18	RAB18, member RAS oncogene family (Rab18)	P35293	19330	2.1	1.3	0.043	0.62	−1.53
Methylcrotonoyl-CoA carboxylase	methylcrotonoyl-Coenzyme A carboxylase 1 (alpha) (Mccc1)	Q99MR8	72039	2.3	1.5	0.023	0.81	−1.51
Profilin	profilin 1 (Pfn1)	Q8CEH8	18643	12.1	8.0	0.026	0.67	−1.51
O-acetyl-ADP-ribose deacetylase	MACRO domain containing 1 (Macrod1)	Q922B1	107227	5.2	3.4	0.030	0.64	−1.50

## Discussion

The purpose of this study was to determine the effects of a 25% KE diet on body weight, body composition, and the skeletal muscle transcriptional, proteomic, and lipidomic responses in aging 72-week-old C57BL/6J mice for nine weeks. We found mice consuming a KE diet remained weight stable, with no changes in FM or LBM, yet the control group increased body weight and fat mass over the course of the study. There were no differences in energy intake between the groups, which agrees with our previous findings in juvenile mice (19). Interestingly, the KE group displayed a unique skeletal muscle transcriptional and proteomic profile compared to the control group yet had a very similar lipidomic profile.

Transcriptional analysis indicated six genes were higher in the KE group compared to CON. Growth Arrest And DNA Damage Inducible Alpha (*Gadd45a)* expression was 1.85-fold higher in KE mice compared to CON. Previous longitudinal data indicates that *Gadd45a* is elevated in aged mouse skeletal muscle during the transition to sarcopenia, which involves progressive muscle atrophy (35). Others have reported that *Gadd45a* expression represents a protective negative feedback response to denervation, which delays the rate of atrophy and myofiber type transition, potentially preserving myofiber type during chronic denervation (36). We found that tenascin-C (*Tnc*) was upregulated 1.44-fold in the KE group, and previous findings have implicated *Tnc* in the formation, maturation, and stabilization of the neuromuscular junction (37). In line with these findings, RUNX Family Transcription Factor 1 (*Runx1)* was up-regulated 1.69-fold in the KE group. Runx1 is required to sustain skeletal muscle by preventing denervated myofibers from undergoing myofibrillar disorganization and autophagy (38). Runx1 ablation results in excessive autophagy during denervation which leads to severe atrophy, suggesting that these findings are protective with regards to age-related autophagy and atrophy of skeletal muscle (39). Other studies have found similar results in aged mice, showing *Gadd45a* and *Runx1* are elevated with a KD, which has been shown to mitigate sarcopenia (10). Another transcript, calponin homology-associated smooth muscle protein (*Chasm)*, which is required for tropomyosin binding, was 1.79-fold lower in KE compared to CON (40). Some have suggested the protein form of CHASM is a discrete marker of Type IIa muscle fibers, which supports the idea that the KE group may be undergoing a transition from Type II to Type I fibers to attenuate muscle denervation induced with aging (41, 42). Decreased calcineurin A (CnA) in our proteomic analysis (−2.82-fold compared to CON) also suggests muscle protection since CnA has been shown to indirectly protect muscle fibers from atrophy by raising the proportion of slow fibers in muscles (43). This occurs, in part, by maintaining peroxisome proliferator-activated receptor-gamma coactivator (PGC-1α) through activation of the myocyte enhancer factor-2 (MEF2) and Nuclear factor of activated T-cells (NFAT) transcription factors (43). *Atrogin-1*, a muscle-specific E3 ubiquitin ligase that is upregulated during atrophy, may contribute to this process because it initiates CnA degradation in cardiomyocytes (44, 45). Therefore, a reduction in CnA could influence the fiber switching that occurs in aging. Taken together, these finding suggest that skeletal muscle may be remodeling toward a slow fiber phenotype in the KE group, potentially protecting against denervation induced muscle fiber loss during sarcopenia. The implications of these findings are not entirely clear and require further exploration to determine if these responses to BD-AcAc_2_ mitigate age-related reductions in skeletal muscle mass and function.

Our results also highlighted the activation of atrophy pathways. For example, we found a small yet significant decrease in Calcium/Calmodulin Dependent Protein Kinase II Beta (*Camk2b)* mRNA expression in KE, which has been identified as a downstream target of p38 mitogen-activated protein kinase alpha (p38α MAPK) and positive regulator of muscle atrophy (46). Furthermore, pharmacological inhibition of CAMK2B activity suppresses denervation-induced muscle atrophy (47). SMAD Family Member 3 (*Smad3)* was also 1.25-fold lower in KE compared to control, which could translate to less atrophy since activation of Smad proteins inhibit the function of *Akt* and the expression of *Atrogin-1* and Muscle-specific RING finger protein 1 (*MuRF1)* by Forkhead box O transcription factors (48). The COP9 singalosome (*Cops4*), which is similar in structure and function to that of the 19S regulatory particle of 26S proteasome, was 2.63-fold higher in KE and has been shown to interact with SCF-type E3 ubiquitin ligases and act as a positive regulator of E3 ubiquitin ligases. Taken together, these findings suggest that the KE may protect against muscle atrophy in aged mice even though skeletal muscle weight was not different between groups, nor were *Atrogin-1* and *MAFbx* mRNA expression. Previous reports indicate that at least one form of KE can attenuate muscle wasting with some diseases in humans (49).

Several transcripts in the KE group were involved in myogenesis, muscle repair, and muscle stem cells. For example, Pim-1 oncogene protein (*Pim1)* was 1.56-fold lower in the current study and *Pim1* is required for proper myogenesis (50). Furthermore, Prominin 1 (*Prom1)* expression was 1.37-fold higher in KE, which is required for stem cell maintenance and activation (51) along with a reduction in the transcripts for MDS1 And EVI1 Complex Locus Protein EV1 (*Mecom)* and Rac Family Small GTPase 3 (*Rac3)*, both of which have been implicated in stem cell control. Our proteomic data support these findings, with Galectin-1 protein content lower in KE mice than CON. Galectin-1 regulates myotube growth in regenerating skeletal muscle (52) and treatment with galectin-1 improves myogenic potential in some mouse models (53). This pathway acts through the Annexin family, and we found that *Annexin A1* and *Annexin 2* mRNA expression were downregulated, in KE mice, which could partially explain our proteomic findings with galectin-1 (52). Annexin A1 affects myoblast fusion causing a slowdown in regeneration of injured muscle but does not cause muscle damage or decrease the ability of injured myofibers to repair (54), while a lack of Annexin 2 results in poor myofiber repair and progressive muscle weakening with age (55). Our proteomic data partially support these findings since Annexin A1 was lower in the KE group compared to CON. PDZ and LIM domain 5 (PDLIM5) plays a positive role in the proliferation and differentiation of skeletal muscle satellite cells, and our proteomic data show it was 1.5-fold lower in KE compared to control (56). The silencing of PDLIM5 increases the nuclear accumulation of differentiation inhibitor (Id2), which inhibits the proliferation and differentiation of myoblasts. Another protein, Fibrillin 1, was higher in KE mice and it influences the skeletal muscle stem cell microenvironment that interacts with bone (57). On the other hand, transgelin was lower in KE mice and previous studies have indicated overexpression or deficiency of transgelin results in significant changes in cell morphology, cytoskeleton, and functional capacity for migration that influence stem cell differentiation (58). Collectively, these data suggest that the KE may affect muscle stem cells or muscle regeneration capacity such that the KE diet is remodeling the muscle stem cell niche in a positive manner, but more research is needed to confirm this speculation.

The ability of the metabolism of ketone bodies to increase insulin sensitivity has been demonstrated previously in the working perfused rat heart (59). This may act through Casitas b-lineage (Cbl) lymphoma, which is involved in regulating the degradation of receptor tyrosine kinases. Genetic mutation of the *Cbl* gene in mice causes a lean phenotype and enhanced peripheral insulin action potentially due to elevated energy expenditure (60, 61). While our previous findings indicate no differences in energy expenditure in younger mice consuming a KE, we found *Cbl* mRNA lower in KE mice compared to control. Interleukin-1 receptor-associated kinase-like 2 (*Irak2*) mRNA expression was also lower in the KE compared to CON and others have shown that *Irak2* reduces high-fat-diet-induced weight gain when inactivated (62, 63). Overall, these findings suggest that BD-AcAc_2_ could be influencing muscle metabolism to improve glucose tolerance and insulin sensitivity. Our findings that control mice gained fat while KE did not gain fat support this possibility and more in-depth analysis with other-omics is warranted.

The lipidomics analysis performed in the current study may also garner insight about the effects of BD-AcAc_2_ on insulin signaling/sensitivity. While BD-AcAc_2_ supplementation appears to have little effect on lipid species within skeletal muscle; the analysis did show that fatty acyl chains in triacylglycerol (TAG) were significantly lower (46%) in the KE group compared to CON. Given our findings on transcripts related to insulin resistance, and that intracellular TAG content is associated with insulin resistance in skeletal muscle, these findings support the overall concept that the KE may improve insulin sensitivity. Additional studies will be required to determine if these effects are direct or if these findings are due to decreased FM (64), or reductions in hepatic lipid content and inflammatory markers (65). Our proteomics data show Acyl-coenzyme A thioesterase 13 (Acot13), a member of the Acyl-CoA thioesterases family that hydrolyze fatty acyl-CoA esters, was lower in KE mice compared to CON (66, 67). Interestingly, mice with whole-body Acot13 deletion have improved glucose homeostasis on a high-fat diet (68). Therefore, lower Acot13 protein content in skeletal muscle of the KE group supports our transcriptomic and lipidomic data.

Our comprehensive characterization of skeletal muscle also suggests that at least some portions of autophagy signaling are affected by BD-AcAc_2_. For example, *disheveled segment polarity protein 2* (*Dvl2*) and TNF receptor associated factor 7 (*Traf7)* were significantly lower in KE compared to CON. Dvl-2 functions as part of the upstream Wnt signal transduction pathway of β-catenin and GSK-3β, and can positively regulate the Wnt signal pathway (69). TRAF7 regulates skeletal muscle through its activity as a ubiquitin ligase and depletion of *Traf7* accelerates myogenesis, in part through downregulation of nuclear factor-κB (NF-κB) activity (70, 71). The downregulation of *Traf7*, along with decreased *Dvl2* in the KE group suggests that BD-AcAc_2_ may reduce cellular stress. Furthermore, profilin 1 protein was lower in the KE group. Increased expression of profilin-1 in vascular smooth muscle cells induces stress fiber formation and causes cardiac hypertrophy and fibrosis by modulating actin polymerization (72, 73). Histone deacetylase 6 (*Hdac6)* expression was also lower, and it controls fusion of autophagosomes to lysosomes (74). Evidence suggests that HDAC6 also plays a role in the eventual clearance of aggresomes, implying a functional connection between HDAC6 and autophagy (74–76). While we can only speculate about the possible role of BD-AcAc_2_ on cellular stress, others have found that a KD attenuates the increase in cellular stress due to aging (10).

Other proteins that were higher in the KE group compared to CON involved elements of the electron transport chain (ETC). For example, crystallin zeta (CryZ), a quinone oxidoreductase and threodoxi-like 1, which controls disulfide oxidoreductate activity was higher in KE compared to CON at the protein level. Another protein elevated in the KE group was voltage-dependent anion channel 1 (VDAC1) which controls the metabolic crosstalk between mitochondria and the cytosol, by regulating the influx and efflux of metabolites, cations, and nucleotides (77). Human VDAC1 deficiency compromises pyruvate oxidation and ATP production (78). Others have shown VDAC1 deficiency causes multiple defects in the mitochondria and ETC complexes in both oxidative and glycolytic striated muscle biopsies. This includes loss of ion homeostasis resulting in irreparable cell injury and concomitantly cell death, primarily through necrosis. Higher expression of electron transfer flavoprotein dehydrogenase (*ETFDH*) mRNA in KE mice suggests improvement of skeletal muscle mitochondria β-oxidation in the ETC (79) while an upregulation of Bcl-2-associated X protein (*Bax)* suggests that the KE may enhance regulation of mitochondrial morphology during apoptosis (80). Given findings that a basal amount of BAX is necessary to maintain energy production *via* aerobic respiration this may be important (81). Taken together, these data indicate that the KE is influencing the ETC, which is similar to what has been reported with a KD in aging mice (10).

Nutritional ketosis has been defined as a metabolic state where β-hydroxybutyrate (β-HB) blood concentrations rise above 0.5 millimole per liter (mM/L) (82, 83). In human clinical trials, circulating ketones such as β-HB reach levels ranging from 1 to 2 mM/L and up to 5 mM/L (6, 84, 85). Comparatively, we (and others) have shown the ability to induce similar levels of ketones in rodents (15, 17, 19, 86). Exogenous ketones offer a way to induce ketone production quickly and efficiently in humans without the need for caloric restriction or a KD and have been well-studied in exercise physiology (11, 12, 87–89). While more research is needed, KE have been suggested as a novel method to treat several diseases, including perturbations that occur in aging (90–94).

A strength of this research is that we took an -omics based approach to determine how a ketone ester influences skeletal muscle, providing multiple avenues for future research. A limitation of this approach is not identifying a specific molecular mechanism by which the KE is acting–although based on our findings it seems the BD-AcAc_2_ is influencing several pathways in skeletal muscle that may be responsible for the physiological and metabolic responses observed. Another limitation is the lack of baseline data on FM and LBM, which was due to COVID-19 shutdowns in the UAB Animal Resources Program. Nonetheless, mice were randomized so this should equally distribute confounding variables at baseline and our previous work in this rodent and dietary model indicates a very homogenous sample in these mice. In addition, body weights of the mice were similar among groups at the start of the study and in our experience with B6 mice, FM of these animals is highly correlated with overall body mass and should not represent a confounding variable for this study. A limitation that muddies the interpretation of our findings are the transcriptional and proteomic analysis being completed in different muscles due to insufficient tissue, so future experiments should include this outcome. The different tissue usage may explain the lack of correlation between our proteomic and mRNA data. Finally, the study is likely underpowered given the number of-omics approaches we utilized and given the lack of previous data on KE and muscle mass at the time of study initiation, we did not have sufficient data for a traditional power calculation.

In conclusion, our findings indicate that a diet containing 25% BD-AcAc2 in male aging B6 mice produces a unique skeletal muscle transcriptional, lipidomic and proteomic signature that influences signaling involved in muscle denervation as well as muscle atrophy and autophagy. Furthermore, KE may improve the satellite cell niche and β-oxidation in the ETC. Our lipidomic data and body composition data align with transcriptional findings that suggest that BD-AcAc2 may play a role in insulin sensitivity. Future studies are needed to examine whether insulin sensitivity is indeed improved in skeletal muscle or whether these findings are related to decreased adiposity and/or fat content in liver.

## Data availability statement

The data presented in the study are deposited in the Open Science Framework repository (https://osf.io/e2yd5/).

## Ethics statement

The animal study was reviewed and approved by UAB Institutional Animal Care and Use Committee (IACUC).

## Author contributions

BR, SD, DS, and EP conceptualized the study. BR and SD conducted the experiments and collected and analyzed the data. NM contributed to the lipidomics data analysis and interpretation. JM contributed to the proteomics data analysis and interpretation. BR wrote the manuscript. All authors edited and approved the final version prior to submission for publication.

## Footnotes

1
https://rosalind.onramp.bio/

## Abbreviations

BD-AcAc_2_, R,S-1,3: butanediol diacetoacetate
KE: ketone ester
LBM: lean body mass
FM: fat mass
KD: ketogenic diet
Tnc: tenascin-C
ER: endoplasmic reticulum
Gadd45a: growth arrest and DNA damage inducible alpha
Runx1: family Transcription Factor 1
Chasm: calponin homology-associated smooth muscle protein
CnA: calcineurin A
PGC-1α: peroxisome proliferator-activated receptor-gamma coactivator
MEF2: myocyte enhancer factor-2
NFAT: nuclear factor of activated T-cells
Atrogin-1/MAFbx: Muscle Atrophy F-box gene
Pim1: pim-1 oncogene protein
Mecom: MDS1 And EVI1 Complex Locus Protein EV1
Camk2b: Calcium/Calmodulin Dependent Protein Kinase II Beta
P38α MAPK: p38 mitogen-activated protein kinases
Irak2: Interleukin-1 receptor-associated kinase-like 2
MuRF1: Muscle-specific RING finger protein 1
Cops9: COP9 singalosome
Dvl1: disheveled segment polarity protein 2 (Dvl2)
Traf7: TNF receptor associated factor 7
Hdac6: histone deacetylase 6
CryZ: crystallin zeta
Bax: bcl-2-associated X protein
VDAC1: voltage-dependent anion channel 1.
